# Combination of optical coherence tomography and near infrared spectroscopy enhances determination of articular cartilage composition and structure

**DOI:** 10.1038/s41598-017-10973-z

**Published:** 2017-09-06

**Authors:** Jaakko K. Sarin, Lassi Rieppo, Harold Brommer, Isaac O. Afara, Simo Saarakkala, Juha Töyräs

**Affiliations:** 1 Department of Applied Physics, University of Eastern Finland, Kuopio, Finland; 2 Diagnostic Imaging Center, Kuopio University Hospital, Kuopio, Finland; 3 Research Unit of Medical Imaging, Physics and Technology, Faculty of Medicine, University of Oulu, Oulu, Finland; 4 Department of Equine Sciences, Faculty of Veterinary Medicine, Utrecht University, Utrecht, Netherlands; 5 Medical Research Center Oulu, Oulu University Hospital and University of Oulu, Oulu, Finland; 6 Department of Diagnostic Radiology, Oulu University Hospital, Oulu, Finland

**Keywords:** Osteoarthritis, Biophotonics

## Abstract

Conventional arthroscopic evaluation of articular cartilage is subjective and poorly reproducible. Therefore, implementation of quantitative diagnostic techniques, such as near infrared spectroscopy (NIRS) and optical coherence tomography (OCT), is essential. Locations (*n* = 44) with various cartilage conditions were selected from mature equine fetlock joints (*n* = 5). These locations and their surroundings were measured with NIRS and OCT (*n* = 530). As a reference, cartilage proteoglycan (PG) and collagen contents, and collagen network organization were determined using quantitative microscopy. Additionally, lesion severity visualized in OCT images was graded with an automatic algorithm according to International Cartilage Research Society (ICRS) scoring system. Artificial neural network with variable selection was then employed to predict cartilage composition in the superficial and deep zones from NIRS data, and the performance of two models, generalized (including all samples) and condition-specific models (based on ICRS-grades), was compared. Spectral data correlated significantly (*p* < 0.002) with PG and collagen contents, and collagen orientation in the superficial and deep zones. The combination of NIRS and OCT provided the most reliable outcome, with condition-specific models having lower prediction errors (9.2%) compared to generalized models (10.4%). Therefore, the results highlight the potential of combining both modalities for comprehensive evaluation of cartilage during arthroscopy.

## Introduction

Articular cartilage is an aneural tissue that enables near frictionless contact between articulating bones. The tissue consists mainly of water, collagen and proteoglycans (PGs)^1^, and due to its limited regenerative capability, it is susceptible to progressive degeneration after trauma and wear. Cartilage defects can result from various reasons and lead to degenerative conditions, such as osteoarthritis (OA) and trauma-induced post-traumatic OA (PTOA), affecting millions of people worldwide^2, 3^. Although these diseases have similar initial stages, including superficial PG loss and disruption of superficial collagen network, PTOA mainly impacts the young, whereas OA mostly the elderly. These alterations in cartilage composition and structure often lead to excessive strains within the tissue, and therefore progressive deterioration. As no cure currently exists for OA, it would be essential to reliably diagnose cartilage injuries and evaluate their surrounding tissue to prevent the initiation and progression of PTOA, e.g. via surgical intervention.

Arthroscopic repair surgery is often performed to treat joint conditions diagnosed via clinical examination, computed tomography (CT) or magnetic resonance imaging (MRI). Due to poor soft tissue contrast in conventional CT images and relatively poor resolution of clinical MRI^4^, arthroscopic examination may reveal previously undetected cartilage lesions. However, clinical assessment of such conditions during conventional arthroscopy is often subjective and poorly reproducible, regardless of the scoring system^5, 6^. Therefore, the implementation of quantitative arthroscopic techniques could enhance the detection of the initial stages of PTOA and, thus, the outcome of the repair surgery.

Multiple techniques, including near infrared spectroscopy (NIRS)^7^, optical coherence tomography (OCT)^8, 9, 10^ and ultrasound^8–10^ have been proposed for arthroscopic evaluation of articular cartilage. The optical techniques, i.e. NIRS and OCT, are complementary as NIRS enables quantitative evaluation of cartilage composition^11, 12^, while OCT provides images with superior resolution compared to CT and MRI. However, scoring of the severity of lesions visualized in these high resolution images is as unreliable as traditional arthroscopic scoring^9, 10^, thus requiring the adaptation of user-independent automatic scoring^9, 10, 13^.

Analysis of near infrared (NIR) spectral data requires application of univariate and multivariate regression techniques, e.g. principal component regression (PCR) and partial least squares regression (PLSR), with the latter being the most commonly adapted^14^. Furthermore, deep learning algorithms, such as multi-layered artificial neural networks (ANN), have performed well with large and complex data due to their ability to model both linear and nonlinear relationships^15, 16^. Additionally, the employment of variable selection methods enables the extraction of the most essential wavelengths and wavebands from variable-rich data, thus resulting in more robust models^17^.

In this study, we hypothesize that the combination of OCT and NIRS provides a comprehensive estimate of cartilage condition, and that ANN can accurately describe the relationship between NIR spectral data and tissue composition and structure. To test these hypotheses, ANN was employed for the first time to model the relationship between cartilage NIR spectroscopic data and its reference parameters, including collagen orientation and PG and collagen contents determined via histological imaging. Furthermore, cartilage lesions visualized in OCT images were evaluated according to the ICRS scoring system with an automatic scoring algorithm^9, 10, 18^. Subsequently, the data were grouped based on their ICRS grades and modelled using ANN, in order to investigate the potential of injury-based (condition-specific) models compared to a generalized model based on all the samples.

## Materials and Methods

**Figure 1:**
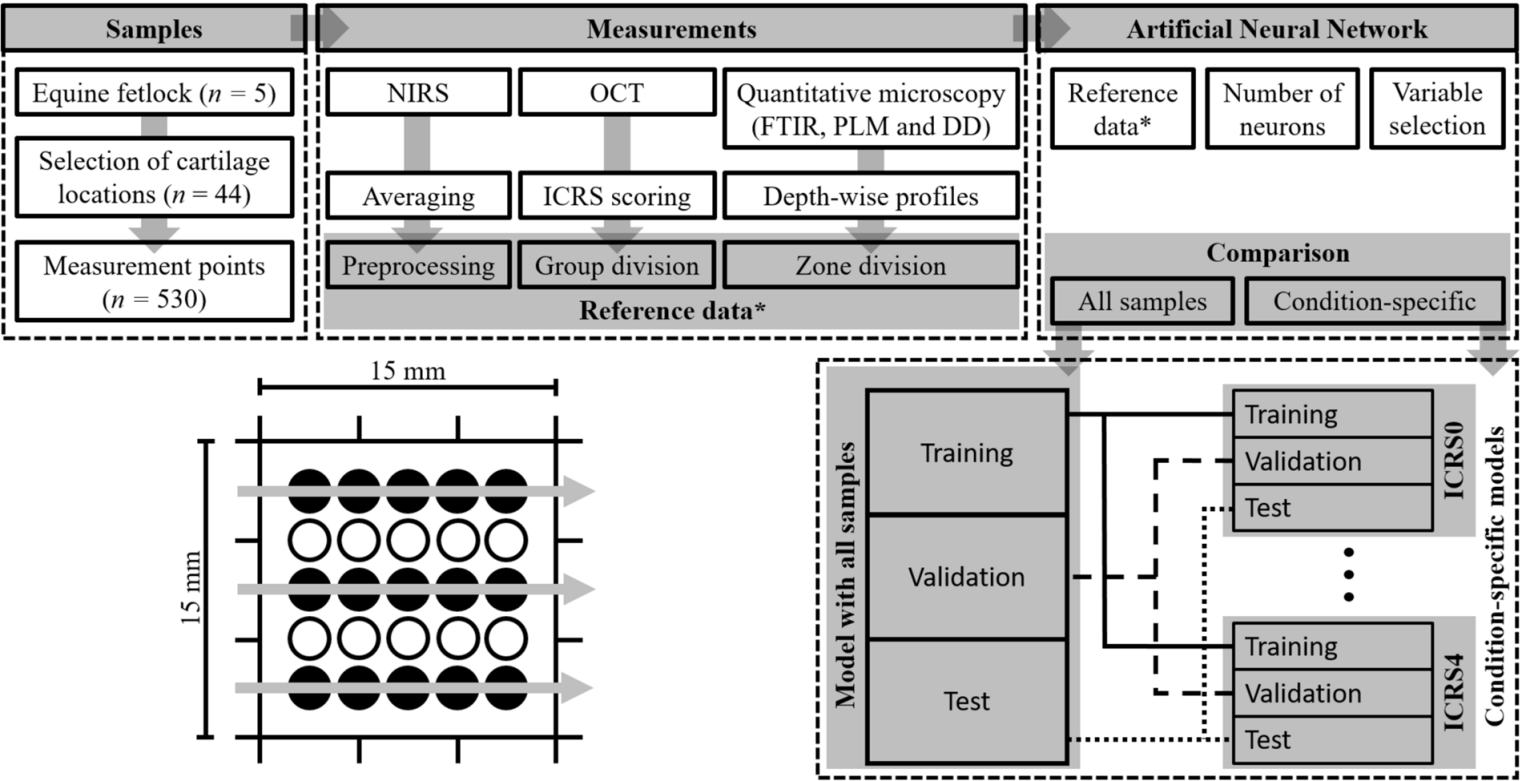
Study design and workflow. During measurements, each cartilage location (*n* = 44) was divided into a 5 × 5 grid (lower left corner) of which the central and outermost lines (filled circles) were measured. After rejecting locations with fully eroded cartilage, 530 measurement points remained. All 530 locations were measured with NIRS, OCT and quantitative microscopy (FTIR, PLM and DD).

Distal metacarpal and proximal 1^st^ phalanx sections were extracted from five limbs of mature equines (*n* = 5), obtained from a slaughterhouse (Equine Slaughterhouse Van de Veen, Nijkerk, Netherlands); thus, no ethical permission for sample collection was required. Cartilage locations (*n* = 44) with various conditions were visually inspected and selected by a board certified equine surgeon (with experience of >500 arthroscopies), and subsequently each location was divided into a grid containing 25 points (15 mm × 15 mm, Fig. 1). The central and outermost lines of the grid (15 points) were measured with NIRS and OCT. Measurement points with fully eroded cartilage were excluded from the study, thus resulting in 530 measurement points. Following NIRS and OCT measurements, each point was subjected to histological analyses by extracting a tissue block for histology. Seven sections (three for both polarized light microscopy (PLM) and digital densitometry (DD), and one for Fourier transform infrared (FTIR) microspectroscopy) were prepared for quantitative microscopy. Due to the large number of measurements required for multivariate modelling and limitations induced by joint anatomy, the NIRS and OCT measurements were not performed arthroscopically.

### Near infrared spectroscopy and spectral preprocessing

The NIR spectra collected in Sarin *et al*.^19^ were utilized in this study. Briefly, the instrumentation consisted of a halogen light source (AvaLight-D(H)S, Avantes BW, Apeldoorn, Netherlands), a spectrometer (AvaSpec-ULS2048XL, Avantes BW) and a diffuse reflectance fibre optic probe. Spectral data in the region 400–1100 nm was acquired with the instrumentation, of which the NIR region (λ = 700–1100 nm) was employed in the analysis as the region consists of stronger overtones of chemical bonds compared to the VIS region^20^. An average of eight consecutive absorbance spectral measurements was collected in three repetitions with reorientation of the probe between rounds (coefficient of variation = 0.82 ± 0.32%^19^). The probe was oriented perpendicular and in contact with sample surface during the measurements. The average spectrum of the three repetitions was smoothed using a third order Savitzky-Golay smoothing filter with a window size of 39 data points (approximately 25 nm) to eliminate any hardware-induced noise. Additionally, 1^st^ and 2^nd^ derivative spectra were calculated as these preprocessing techniques are known to accentuate subtle absorption peaks^21^, therefore improving the prediction accuracy and decreasing errors of calibration models. Furthermore, the effectiveness of normalization methods, i.e. multiplicative scatter correction (MSC) and standard normal variate (SNV), were investigated.

### Optical coherence tomography and automatic scoring algorithm

The NIRS measurement points were imaged using OCT (wavelength 1305 ± 55 nm, axial resolution <20 µm, lateral resolution 25–60 µm; Ilumien PCI Optimization System, St. Jude Medical, St. Paul, MN, USA) to detect and evaluate possible cartilage lesions^22^. An OCT imaging catheter was orientated parallel and as close to the cartilage surface as possible without making contact. The samples were submerged in phosphate-buffered saline during the imaging.

A customized automatic algorithm was utilized for scoring the lesions visualized in the OCT images according to ICRS scoring system^9, 10, 18^. The algorithm, introduced by te Moller *et al*.^18^, was refined by introducing adaptive smoothing, thus enhancing the detection of cartilage-bone interface. In summary, the imaging catheter (*d* = 0.9 mm) is detected and removed from the OCT image (2048 × 2048 pixels). The catheter size is later utilized as a scaling factor to quantify cartilage surface roughness. Next, a region of interest (width = 2 mm), matching the size of the fibre optic probe applied in the NIRS measurement, is chosen. This is followed by the detection of cartilage surface and the interface between non-calcified and calcified cartilage, in order to determine OCT roughness index (ORI)^23^, i.e. articular surface roughness, and cartilage thickness. Thresholds for ORI (8 μm) and cartilage loss (8%) were applied, as in our previous studies^9, 10, 18^, to differentiate between ICRS grades 0–1 and 1–2, respectively. According to the International Cartilage Research Society scoring guidelines^24^, defects extending deeper than 50% of cartilage thickness are categorized as grade 3, and defects extending into subchondral bone as grade 4.

### Histology

Osteochondral samples extracted from the measurement locations were fixed in formalin, decalcified in EDTA and embedded in paraffin blocks. Sections (*n* = 7) were cut with a microtome along each measurement line for the histological imaging modalities, i.e. FTIR microspectroscopy (*n* = 1), PLM (*n* = 3) and DD (*n* = 3). The section thicknesses for the imaging modalities were 5 μm, 5 μm and 3 μm, respectively.

### Fourier transform infrared (FTIR) microspectroscopy

Collagen and PG distributions were determined from the histological sections via FTIR microspectroscopy by mapping 500-μm-wide areas covering the full cartilage thickness in mid infrared (MIR) region. Same sized areas were also measured with PLM and DD. FTIR measurements were conducted with the Thermo iN10 FT-IR microscope (Thermo Nicolet Corporation, Madison, WI, USA) in transmission mode using a spectral resolution of 4 cm^−1^ and pixel size of 25 × 25 μm^2^. Four repetitive measurements per pixel were acquired and averaged. The collagen and PG contents were determined as the integrated area of the amide I peak (1584–1720 cm^−1^) and the carbohydrate region (984–1140 cm^−1^), respectively^25^.

### Polarized light microscopy

The orientation and birefringence of collagen in the samples were determined by imaging with Abrio PLM system (CRi, Inc., Woburn, MA, USA) mounted on a conventional light microscope (Nikon Diaphot TMD, Nikon, Inc., Shinagawa, Tokyo, Japan). The Abrio system consists of a green bandpass filter, a circular polarizer, and a computer-controlled analyser composed of two liquid crystal polarizers and a CCD camera. All specimens were imaged at identical orientation with a 4.0x objective, which resulted in a pixel size of 2.53 × 2.53 μm^2^. In the orientation images, 0 degrees corresponds to the orientation parallel to cartilage surface and 90 degrees perpendicular to cartilage surface.

### Digital densitometry

The 3 µm sections were stained with Safranin-O and measured with quantitative DD system to determine PG distribution^26^. The system consists of a light microscope (Nikon Microphot-FXA, Nikon Co., Tokyo, Japan), equipped with a monochromatic light source and a 12-bit CCD camera (ORCA-ER, Hamamatsu Photonics K.K., Hamamatsu, Japan). The system was calibrated with neutral density filters (Schott, Mainz, Germany) covering optical density (OD) range from 0 to 3.0. The samples were imaged with a 4.0x objective resulting in a pixel size of 1.56 × 1.56 μm^2^.

### Determination of cartilage zones

The interface between uncalcified and calcified cartilage was manually determined from the histological (Safranin-O) images. Additionally, the depth-wise profiles of the reference parameters were divided into two zones, with the first defined as the traditional superficial zone and the second as a combination of the traditional intermediate and deep zones. In the context of this study, these zones are referred to as superficial and deep zones. The interface between the superficial and deep zones was determined as the minimum point of the birefringence profile, indicating random orientation of collagen fibers^26^. The minimum superficial zone thickness was 12.5 µm, which was observed in severe cartilage defects (ICRS2).

### Artificial Neural Network

ANN models were created to predict cartilage composition and collagen orientation from the preprocessed NIR spectral data. For ANN model training, one hidden layer with a maximum of 10 neurons was chosen to minimize overtraining, which can result from too many neurons and inputs. Additionally, an input variable selection (IVS) technique, i.e. filter-based forward variable selection, was utilized to discover the most important variables^17^. In this IVS technique, the single most reliable wavelength is first determined, followed by iteratively identifying the next most reliable variable. The IVS was terminated after the most relevant 200 variables were determined. The Levenberg-Marquardt backpropagation algorithm was chosen for ANN training due to its optimization efficiency in modelling complex data. The transfer functions in the hidden and output layers were hyperbolic tangent and linear functions, respectively. To ensure reproducible results, the effect of initial weights was tested and recorded during model building. The sizes of training, validation and test groups were 60%, 30% and 10% of all measurement points, respectively. The validation and test groups were determined by randomly selecting measurement points (30% and 10%, respectively) within each ICRS grade group formed using the automatic scoring algorithm. The ranges of the reference parameters in the validation and test groups were within the range of the training group. ANN training was terminated when the validation error did not decrease in six successive iterations. The optimal model was chosen based on the root mean square error (RMSE) of the test group.

Two separate protocols were adapted to investigate the potential of condition-specific models. In the 1^st^ protocol, an ANN model was created with all the samples (generalized model). In the 2^nd^ protocol, separate training, validation and test groups were created for each ICRS grade (condition-specific models). Furthermore, these groups were subgroups of the training, validation and test groups of the 1^st^ protocol (Fig. 1). Similar optimization was performed in the development of the models in both protocols. Additionally, to compare the various preprocessing methods and the effect of IVS and condition-specific division, the normalized root mean square error (NRMSE) was determined as RMSE divided by the range of the reference parameter. ANN modelling was performed in MATLAB (Matlab R2016b, MathWorks Inc., Natick, MA, USA) using the neural network toolbox (Version 9.1).

### Statistics

The statistical significance of the test group (*n* = 53) correlation was evaluated using the two-tailed Pearson’s correlation analysis by comparing the reference values to the values predicted by the ANN models. The Kruskal-Wallis test (*n* = 530) was employed to investigate the statistical significance of difference between the reference parameter values in the ICRS-scoring based groups. All statistical analyses were conducted in IBM SPSS statistics software (Version 23, SPSS Inc., Chicago, USA).

Data of the current study is available from the corresponding author on reasonable request.

## Results

**Figure 2:**
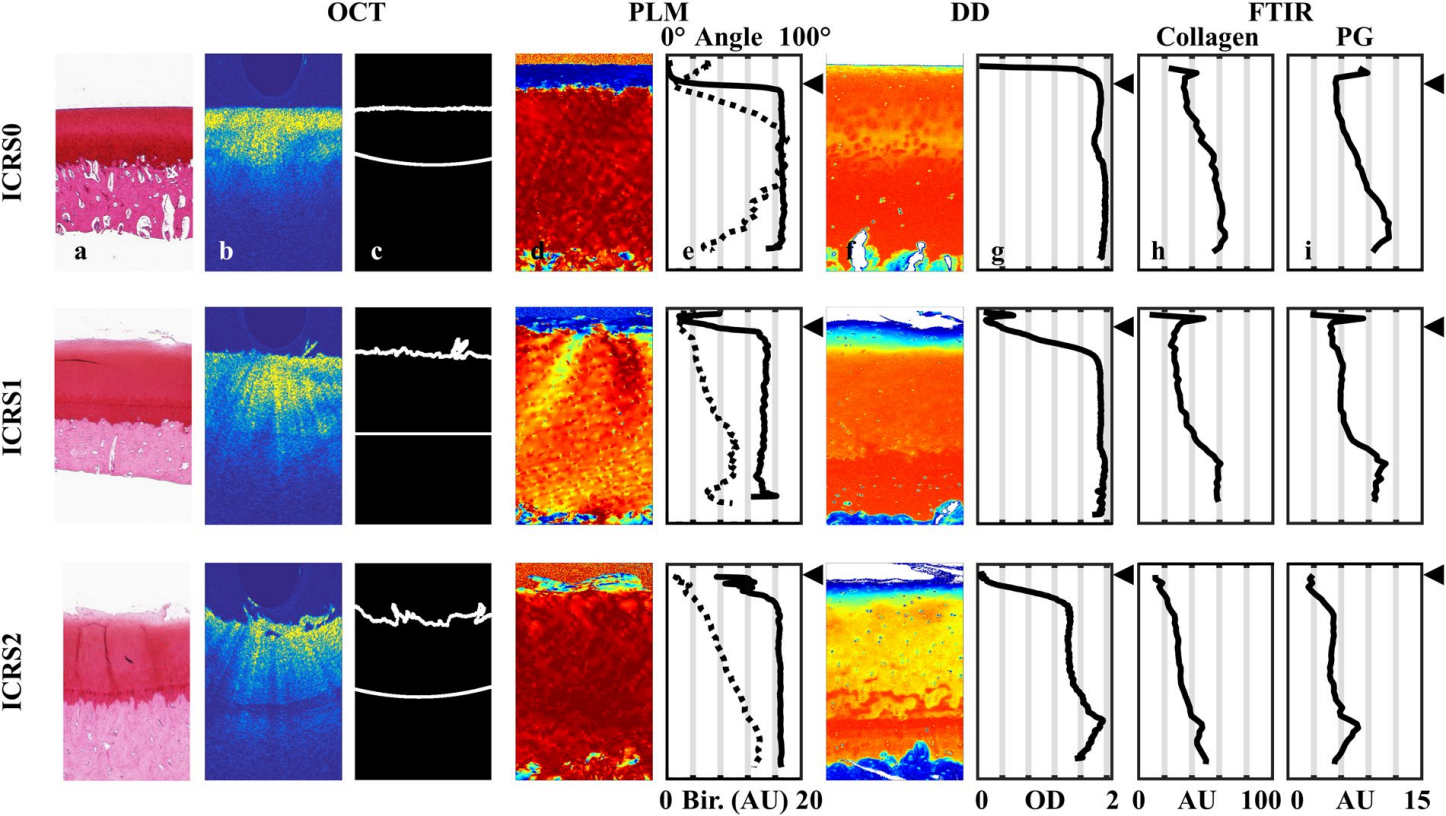
Representative examples of ICRS0, ICRS1 and ICRS2 grades visualized using light microscopy (**a**, Safranin-O stained) and OCT (**b**), along with the cartilage surface and cartilage-subchondral bone interface (**c**) automatically segmented from the OCT image. Followed by PLM (**d**,**e**) images with depth-wise collagen orientation angle (solid line) and birefringence (Bir., dashed line) profiles, DD (**f**,**g**) images with depth-wise OD profile, and FTIR-based (**h**,**i**) depth-wise profiles of collagen and PG contents. The interface between the superficial and deep zones is presented with the black triangle.

**Figure 3:**
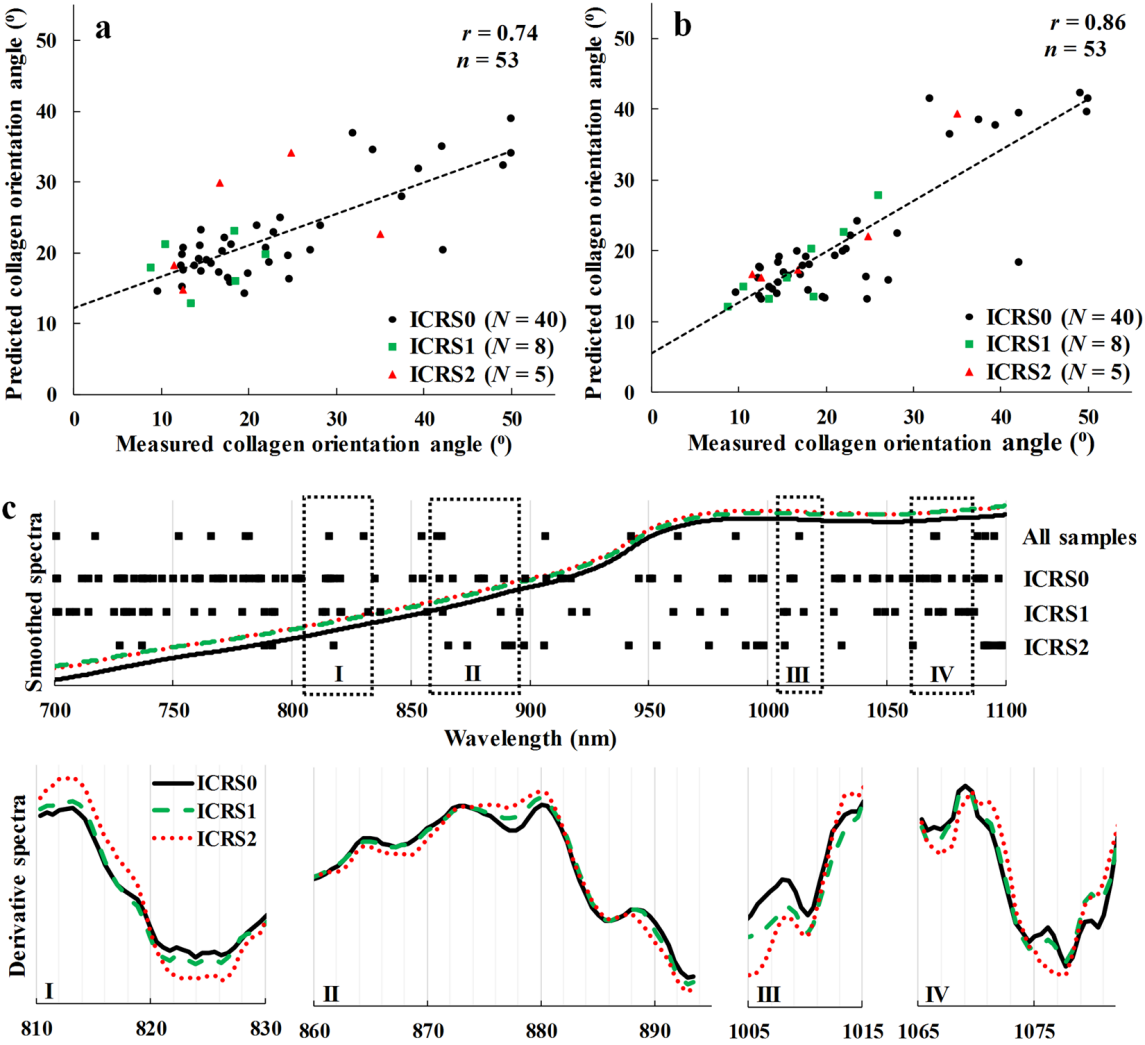
Prediction of collagen orientation angle in the superficial zone with a generalized model (**a**) and with the three condition-specific models (**b**). The average smoothed spectra of ICRS0, ICRS1 and ICRS2 (**c**) with the selected wavelengths (black regions) of IVS for each condition-specific model of superficial collagen orientation angle. Furthermore, differences in the spectra of the ICRS groups are highlighted in the 2^nd^ derivative average spectra (y-axes not in scale) within the mutual wavelength regions (I-IV).

Distinct differences in the histology, OCT, and microscopy images (Fig. 2) were observed between samples from different ICRS groups as well as average NIR spectra (Fig. 3c), thus highlighting the sensitivity of these optical techniques. Data from the 700–1100 nm spectral region correlated (*p* < 0.0002) with collagen orientation, collagen content, and PG content (Table 1, Fig. 3). The average NRMSEs indicated that combination of only spectral smoothing with 1–8 neurons was the most reliable approach (11.7%), when compared to SNV (11.8%) and MSC (11.9%) normalization, and 1^st^ derivative (12.2%) and 2^nd^ derivative (12.5%) preprocessing. Furthermore, both the adaptation of IVS (10.4%) and the condition-specific models combined with IVS (9.2%) systematically decreased the average NRMSE. The average thickness values of the superficial and deep zones were 77 ± 42 µm and 698 ± 215 µm, respectively.

**Table 1:** Average, standard deviation (SD) and range for the measured reference parameters along with correlation and RMSE values for the test group.

		Mean ± SD (Range)	All samples			ICRS-based division		
			*r*	RMSE	*p*	*r*	RMSE	*p*
**Collagen orientation angle** (^π^)	Superficial	23.2 ± 12.9 (5.6–74.4)	0.74	7.37	<0.0001	0.86	5.91	<0.0001
	Deep	77.1 ± 7.6 (39.2–87.5)	0.52	5.72	<0.0001	0.50	5.85	0.0001
**Collagen content** (**AU**)	Superficial	21.6 ± 9.2 (1.4–48.3)	0.62	5.84	<0.0001	0.72	5.18	<0.0001
	Deep	34.7 ± 9.2 (12.7–68.0)	0.65	5.59	<0.0001	0.78	4.81	<0.0001
**PG content** (**AU**)	Superficial	5.34 ± 2.41 (0.15–18.99)	0.42	1.24	0.0018	0.69	0.97	<0.0001
	Deep	6.47 ± 1.99 (0.50–15.58)	0.70	0.99	<0.0001	0.77	0.89	<0.0001
**PG content** (**OD**)	Superficial	1.02 ± 0.49 (0.09–1.82)	0.76	0.30	<0.0001	0.78	0.28	<0.0001
	Deep	1.79 ± 0.23 (0.28–2.00)	0.63	0.10	<0.0001	0.82	0.08	<0.0001

**Figure 4:**
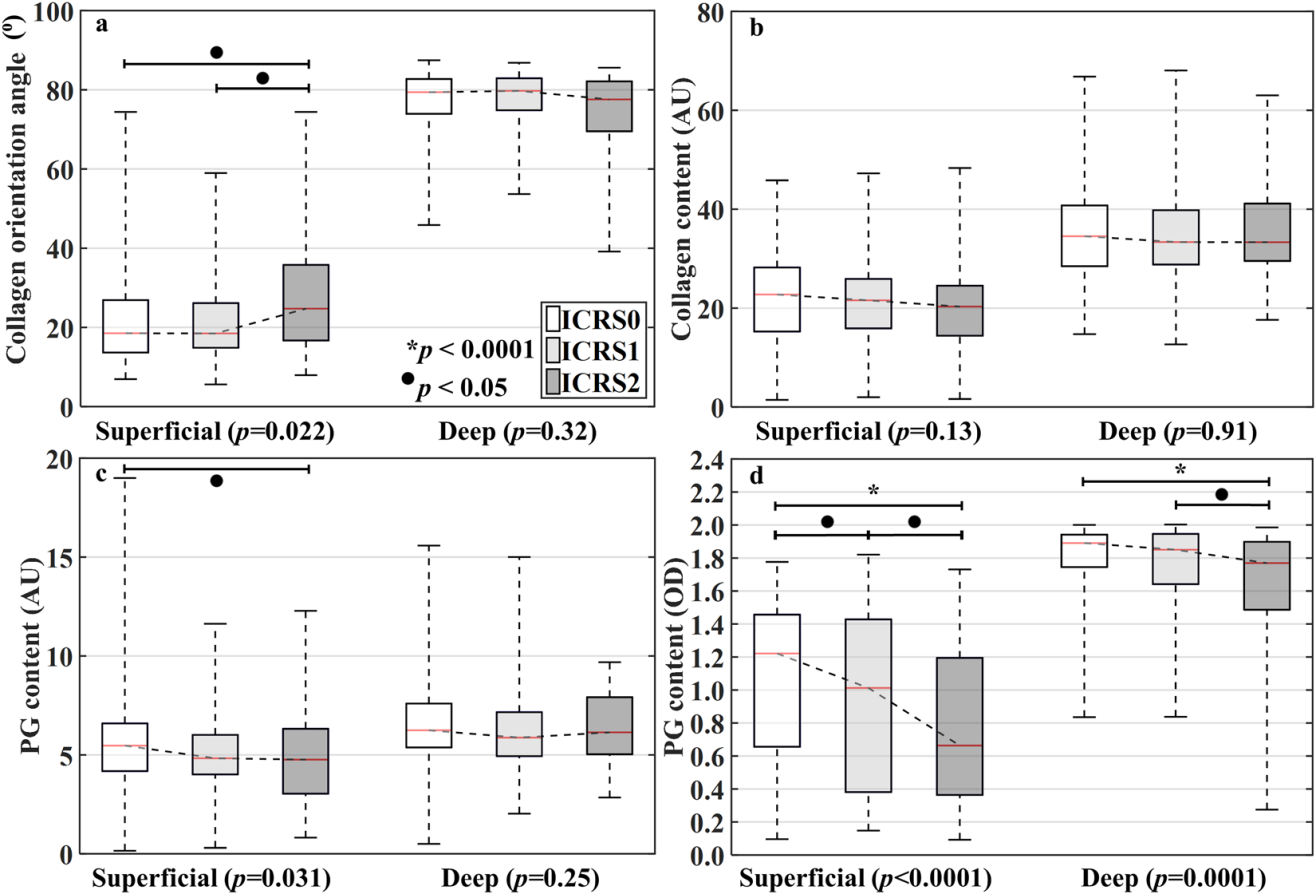
Boxplot showing the measured (*n* = 530) distribution of collagen orientation angle (**a**), collagen content (**b**), and PG content (**c,d**) with respect to the ICRS grades determined with the automated algorithm (median, 25% and 75% quartiles and range).

The average superficial collagen and PG contents decreased with increasing ICRS grade, whereas the collagen orientation angle in respect to cartilage surface increased (Fig. 4). These alterations in tissue composition and structure decrease the capability of the tissue to bear loads, therefore potentially leading to excessive strains and, thus, progressive damage. The severity of cartilage lesions observed in the samples was ICRS0 (*n* = 318), ICRS1 (*n* = 159) and ICRS2 (*n* = 53).

## Discussion

In this study, we combine for the first time imaging and non-imaging optical modalities, i.e. OCT and NIRS, respectively, for evaluation of cartilage condition. The OCT-based scoring of cartilage lesions enhanced the prediction of compositional and structural properties from NIR spectral data, thus demonstrating the potential of combination of these modalities for arthroscopic evaluation of articular cartilage. The relationship between cartilage composition and spectral data in the NIR and MIR regions^11, 12^ has been established; however, no study has investigated the relationship between NIR spectral data and collagen orientation. Furthermore, no previous spectroscopic studies of articular cartilage have utilized ANNs for spectral analyses. We successfully employed ANN to investigate the relationship between articular cartilage NIR spectral data and its composition and structure.

The employed spectral region (700–1100 nm), coupled with the adaptation of ANN modelling, resulted in significant (*p* < 0.002) correlations between the NIR spectral data and collagen content and orientation, and PG content. These relations arise from the chemical bonds of cartilage constituents (collagen and PG)^27^, mainly the second and third overtone vibrations of CH, NH and OH. The second and third overtones of NH stretching vibration appear in the regions of 950–1100 nm and 775–850 nm, respectively, whereas second overtone OH stretching vibration appears at 950–1100 nm and third overtone CH stretching vibration at 850–950 nm^20^. Due to high water content (65–80%) of articular cartilage, OH absorption is the most pronounced in the spectrum with evident peak at 970 nm. The utilized spectral region has been previously shown to correlate with PG content^28^. However, the relation of collagen orientation with spectroscopic data has not been previously investigated. This relation is expected to arise from the birefringence property of cartilage. Furthermore, the average absorbance spectrum of visually healthy cartilage (ICRS0) was lower compared to damaged cartilage (Fig. 3c), which may indicate that more light is reflected back from the intact superficial collagen network due to the heterogeneous structure of cartilage. Thus, the reflected light was expected to include compositional information on superficial cartilage. The reflection effect was also expected to be wavelength-specific along with the slightly varying penetration depth^29^. Unfortunately, no physical model has been developed for cartilage to investigate wavelength-specific penetration into the tissue or the role of cartilage structure^29^ on light penetration. Due to this limitation, we opted for an IVS technique to determine the most informative wavelengths for each parameter. Moreover, the relative absorbance has also been associated with cartilage thickness due to the differences in optical pathlength^19^; therefore, relative absorbance is most probably affected by both parameters: cartilage thickness and superficial collagen network.

Multivariate regression techniques, such as PCR and PLSR, have been widely utilized for analyses of articular cartilage spectral data, whereas deep learning algorithms, such as ANN, have never been applied, despite encouraging results achieved in other spectroscopic applications^16, 30, 31^. The necessity of a large number of samples for accurate training of ANN models has arguably limited its application in the analyses of cartilage spectra. As a result of the high number of spectral measurements in this study and the positive outcomes of several spectroscopic studies^15, 30^, we opted for ANN over the conventional multivariate regression techniques. ANN has consistently outperformed linear operators, i.e. PCR and PLSR, arguably due to its ability to model non-linearity^32^, which in the case of linear operators cannot be completely accounted for with mathematical preprocessing techniques^33^. This is supported by our findings that spectral smoothing operation alone yielded models with the lowest predictions errors, compared to models combining smoothing and normalization techniques or derivative preprocessing. Similar finding has been presented by Ni *et al*.^31^. Although the variation in NRMSE values is minimal, the elimination of redundant preprocessing enables more rapid data processing during *in vivo* applications.

The filter-based IVS iteratively identified the most relevant wavelengths, and thereby systematically decreased the prediction errors of the test group and computational time in the model development. The number of the most relevant variables was limited to 200 as no substantial improvement was observed by increasing the variable number in pretesting of the IVS. Additionally, the spectral region employed in this study was limited to short NIR spectral range (700–1100 nm); therefore, the application of the whole NIR range (700–2500 nm) could enhance the reliability of the prediction. However, this range includes relatively higher absorbance overtones, and suffers from the dominating absorbance of water.

The relationships between ICRS grades and cartilage properties obtained in this study are in agreement with previously observed variations^34, 35^, therefore supporting the reliability of the scoring. The quantification of PG amount varied slightly between the two methods, i.e. DD and FTIR, as Safranin-O staining quantifies only the sulfated PGs, whereas the carbohydrate region of FTIR also includes the non-sulfated PGs and glycoproteins^36^. With FTIR, the measurements were limited to one section compared to three of PLM and DD; thus, possible variations in the section thickness could not be similarly eliminated with averaging. Determination of the PG and collagen contents was based on the integrated area of well-established spectral peaks^25^. Although derivative preprocessing has been shown to improve the outcome of FTIR analysis, this preprocessing technique increases spectral noise and requires high spectral resolution, thus increasing the acquisition times. Nevertheless, in the NIRS analysis of this study, the OCT-based ICRS scoring enabled division of the cartilage samples into subgroups, which enhanced the prediction accuracy and, thus, the reliability of the ANN models. The combination of OCT and NIRS could result in less-subjective evaluation of severe chondral defects by visualization, i.e. using OCT, and also the detection of compromised cartilage surrounding the lesion, e.g. at the initial stages of PTOA, prior to any visible signs of tissue alterations. Furthermore, the non-overlapping wavelength regions of OCT and NIRS enable simultaneous data acquisition and diagnosis.

The contribution of subchondral bone to the NIR data could not be accounted for in this study as the depth-wise optical properties of articular cartilage have not yet been fully investigated. However, the NIR region has a superior penetration depth compared to MIR region^29^, therefore not restricting the analysis to the superficial layer of cartilage. Furthermore, applying even shorter wavelengths, i.e. visible region, could enable the evaluation of subchondral bone after quantifying the optical properties of cartilage. In few cases of severe cartilage defects, the thin superficial zone included a part of the traditional intermediate zone. However, no effect was observed on the prediction performance of the ANN models in these cases, thus suggesting that NIRS is reliable for assessing the orientation of collagen fibrils. No biochemical assays were conducted in this study due to the necessity for depth-wise determination of cartilage composition; however, Afara *et al*.^12^ reported strong correlations between cartilage biochemical composition and NIR spectra by utilizing a similar spectral range.

In conclusion, ANN can reliably model the relationship between NIR spectral data and cartilage composition or structure with minimal preprocessing. Furthermore, condition-specific classification of cartilage samples based on OCT further enhanced the prediction capability of NIRS, therefore highlighting the importance of the combination of NIRS and OCT for arthroscopic applications.
